# A prognostically meaningful definition of non-dilated left ventricular cardiomyopathy

**DOI:** 10.1007/s10554-025-03474-y

**Published:** 2025-07-31

**Authors:** Alberto Aimo, Ignazio Alessio Gueli, Bianca Alderotti, Irina Bellisario, Giancarlo Todiere, Chrysanthos Grigoratos, Carmelo De Gori, Alberto Clemente, Giorgia Panichella, Giuseppe Vergaro, Alberto Giannoni, Nicoletta Botto, Simona Vittorini, Claudio Passino, Giovanni Donato Aquaro, Filippo Cademartiri, Michele Emdin, Andrea Barison

**Affiliations:** 1 Interdisciplinary Center for Health Sciences, Scuola Superiore Sant’Anna, Pisa, Italy; 2 Cardiology Division, Fondazione Toscana Gabriele Monasterio, Piazza Martiri della Libertà 33, 56124 Pisa, Italy; 3 Emergency Medicine, Azienda ospedaliera Universitaria Pisana, Pisa, Italy; 4 Department of Neuroscience, Imaging and Clinical Sciences, University of Chieti, Chieti, Italy; 5 Radiology Department, Fondazione Toscana Gabriele Monasterio, Pisa, Italy; 6 Cardiology Division, Careggi University Hospital, Florence, Italy; 7 Academic Radiology Unit, Department of Surgical, Medical and Molecular Pathology and Critical Area, University of Pisa, Pisa, Italy

**Keywords:** Cardiomyopathy, Left ventricle, NDLVC, Diagnosis, Prognosis

## Abstract

Non-dilated left ventricular cardiomyopathy (NDLVC) has been defined as non-ischemic LV scarring and/or fatty replacement and/or hypokinesia, without LV dilation. We tried to identify specific criteria for LV dilation and dysfunction to implement this definition. We identified all non-ischemic cardiomyopathy patients undergoing a cardiovascular magnetic resonance scan from 2012 to 2022 with LV ejection fraction (LVEF) < 55% and/or non-ischemic late gadolinium enhancement (LGE) and/or fatty replacement, and without specific etiologies. The primary endpoint was a composite of all-cause death, sustained ventricular tachycardia or fibrillation. The cohort included 388 patients (32% women, median age 55 years [interquartile range 43–63]). Over 4.3 years (1.9-7.0), 59 patients (15%) developed a primary endpoint event. The risk increased exponentially with LVEDVi values, with inflection points approaching the upper reference limit of LVEDVi (< 96 mL/m^2^ in women, < 105 mL/m^2^ in men). Using these criteria, we identified NDLVC in 237 patients (61%). Among them, LVEF was the only univariate predictor of outcome. Patients with LVEF ≥ 45% (*n* = 212) or ≥ 40% (*n* = 223) had a longer survival than those with LVEF < 45% or < 40% (*p* = 0.025 and *p* < 0.001, respectively). NDLVC patients, identified by non-ischemic LV scarring and/or fatty replacement and/or hypokinesia and LVEDVi < 96 mL/m^2^ (women) or < 105 mL/m^2^ (men), have a lower risk of death or ventricular arrhythmias than patients with dilated cardiomyopathy. LVEF < 45% or < 40% further stratified outcome. The definition of NDLVC could incorporate these LVEDVi and LVEF cut-points to identify a population of patients with a homogeneous risk of death or ventricular arrhythmias.

**Supplementary Information:**

The online version contains supplementary material available at 10.1007/s10554-025-03474-y.

## Background

The recent European Society of Cardiology Guidelines on cardiomyopathy introduced a working diagnosis of non-dilated left ventricular cardiomyopathy (NDLCV), defined as non-ischemic LV scarring or fatty replacement in the absence of LV enlargement, occurring alongside global or regional wall motion irregularities, or isolated global LV hypokinesia without scarring (identified by late gadolinium enhancement [LGE] on cardiovascular magnetic resonance [CMR]), not solely attributable to abnormal loading conditions (like hypertension or valvular disease) or coronary artery disease [1]. This new entity thus encompasses conditions ranging from hypokinetic non-dilated cardiomyopathy to arrhythmogenic LV or left-dominant cardiomyopathy, and to other borderline phenotypes characterized by LV systolic dysfunction or tissue changes without LV dilation, not strictly fulfilling diagnostic criteria for dilated cardiomyopathy (DCM) or arrhythmogenic right ventricular cardiomyopathy (ARVC) [1]. The proposed definition of NDLVC leaves certain aspects unclear, including the specific criteria for LV dilation and whether factors such as LV systolic dysfunction, RV involvement, or the presence of a likely pathogenic or pathogenic (LP/P) gene variant should be considered. Clarifying the prognostic value of these variables may help determine whether they should be incorporated into the definition of NDLVC and which thresholds should be used to identify a patient population with consistent outcomes.

## Methods

### Patient population

From the electronic health records of a tertiary referral center (Fondazione Toscana Gabriele Monasterio (FTGM, Pisa and Massa, Italy) we retrospectively identified all adult patients referred for non-ischemic cardiomyopathy who had undergone a CMR scan from 2012 to 2022 (7,643 patients). We excluded patients with cardiac dysfunction attributable to abnormal loading conditions or coronary artery disease, hypertrophic cardiomyopathy or any specific etiology (including amyloidosis, sarcoidosis, acute myocarditis, cardiac tumors), and patients with ARVC. In agreement with the current definition of NDLVC [1], we then selected all patients who had left ventricular ejection fraction (LVEF) < 55% and/or LV scarring with a non-ischemic pattern (i.e., subepicardial or midwall LGE) and/or fatty replacement (1,250 patients). The 55% LVEF threshold was selected as a conservative cut-off for systolic dysfunction, close to the 56% cut-off defined by a European Association of Cardiovascular Imaging consensus paper [2]. Among patients with no specific etiology, LVEF < 55% and/or LV scarring with a non-ischemic pattern and/or fatty replacement, 388 patients had a complete genetic testing for cardiomyopathy-associated genes (Supplemental Fig. 1). These last patients had undergone a complete clinical evaluation and genetic analysis (next-generation sequencing, Illumina’s NextSeq™ 500 instrument, Twist Custom Panel, Cardio-v3 [Diatech] V3.0, *n* = 202 genes) as part of their diagnostic workup, together with the CMR scan.

The study conformed to the Declaration of Helsinki and was approved by the Institutional Review Board. Patients provided written informed consent for the use of their data for research purposes.

### Cardiovascular magnetic resonance

All participants underwent CMR examination on a 1.5 T scanner (Signa CVi from 2012 to 2017, Signa Artist from 2018 to 2022, GE-Healthcare, Milwaukee, USA). CMR scans were interpreted by readers (A.B. and I.G.) blinded to all other patient data. Steady-state free precession sequences were used to acquire cine images in standard long- and short-axis views.

LGE images were acquired 10–15 min following a bolus administration of 0.2 mmol/kg gadolinium contrast agent. LV and RV volumes and function were measured using the standard volumetric technique from the cine short axis stack and indexed to body surface area [3]. Regional wall motion abnormalities were visually assessed and described using a 17-segment model of the LV [4]. Fatty infiltration was evaluated by the presence of areas surrounded by banding artefacts within the LV and/or RV myocardium in SSFP images, confirmed in at least two orthogonal planes [5]. LGE presence was defined by the identification of areas of increased signal intensity confirmed in two orthogonal planes or after phase/frequency direction swapping. LGE pattern was visually classified as subepicardial, intramyocardial, subendocardial or transmural. Subtle areas of LGE confined to the RV insertion points were not deemed pathological [6]. Semiquantitative assessment of LGE extension was calculated as the total number of LV segments with LGE. LGE extent was also quantified in the short-axis slices using manually drawn endocardial and epicardial borders and a semi-automated threshold of 6 standard deviations above a normal reference region of interest, and expressed as a percentage of total LV mass [7].

As stated above, patients with exclusive RV involvement (i.e., ARVC) were not included in the study cohort. Definite biventricular ACM was defined according to the European Task Force criteria: considering that all patients presented by definition at least one minor morpho-functional criterion (i.e. LV systolic dysfunction and/or LGE), biventricular ACM was defined both by the presence of at least another minor morpho-functional criterion affecting the RV and by the total number of biventricular criteria (i.e. at least 2 major, 1 major + 2 minor or 4 minor criteria) [8].

### Follow-up and study endpoints

Patients were followed over time in a dedicated outpatient clinic and managed according to ESC Guideline recommendations [9–11]. Independent interviewers obtained information from electronic health records or from patients, cardiologists or general practitioners. The primary endpoint was a composite of all-cause death, sustained ventricular tachycardia (VT) or ventricular fibrillation (VF). The secondary endpoint was a composite of cardiac death, sustained VT or VF.

### Statistical analysis

Statistical analysis was performed using SPSS Statistics version 24.0 (Armonk, NY, USA) and R (version 4.2.2). Normal distribution was assessed through the Shapiro-Wilk test. Continuous variables with normal distribution were presented as mean ± standard deviation, and those with non-normal distribution as median and interquartile range. Categorical variables were presented as absolute numbers and percentages and compared by the Chi-square test with Yates correction. Mean differences between groups were evaluated through the Mann-Whitney’s U test. Cubic spline interpolation (with 4 degrees of freedom) was carried out to represent the changes in risk according to left ventricular end-diastolic volume index (LVEDVi). Kaplan-Meier curves with log-rank calculation were derived. The proportional hazard assumption was checked. Predictors of outcome were searched through univariable Cox regression analysis. p values < 0.05 were considered as significant.

## Results

### Study population

The whole cohort included 388 patients with LVEF < 55% and/or LV scarring with a non-ischemic pattern and/or fatty replacement, and no specific etiology. As reported in Table 1, women accounted for 32% of the population, and median age was 55 years (interquartile range 43–63 years). A likely pathogenic or pathogenic (LP/P) variant was found in 24% of patients, and 10% had a LP/P variant in a desmosomal gene. The specific variants are reported in Supplemental Table 1.

**Table 1:** Patient characteristics

	Patients *n* = 388
Age (years)	55 (43–63)
Female sex, n (%)	125 (32)
Family history of SCD, n (%)	118 (31)
NT-proBNP (ng/L)	169 (63–506)
NYHA class I/II, n (%)	147/241 (38/62)
Hypertension, n (%)	93 (24)
Diabetes, n (%)	31 (8)
*Gene testing*	
Negative/VUS/LP or P variant, n (%)	225/72/92 (58/19/24)
LP or P variant in a desmosomal gene, n (%)	40 (10)
*CMR findings*	
LVEDVi (mL/m^2^)	94 (78–118)
LVEF (%)	51 (41–60)
LVMI (g/m^2^)	69 (59–83)
LV wall motion abnormalities, n (%)	218 (56)
LGE presence in the LV, n (%)	294 (76)
n of LV segments with LGE, n	2 (1–5)
Percent LGE mass (% of LV mass)	5 (0–10)
Fatty replacement in the LV, n (%)	163 (42)
n of LV segments with fatty replacement, n	1 (0–2)
RVEDVi (mL/m^2^)	82 (69–96)
Moderate-to-severe RV dilation, n (%)	6 (2)
RV wall motion abnormalities, n (%)	160 (41)
RVEF (%)	57 (50–63)
RV LGE, n (%)	46 (12)
Fatty replacement in the RV, n (%)	124 (32)

Percentages were calculated out of patients with available data. CMR, cardiovascular magnetic resonance; LGE, late gadolinium enhancement; LP/P, likely pathogenic or pathogenic; LVEDVi, left ventricular end-diastolic volume index; LVEF, left ventricular ejection fraction; LVMI, left ventricular mass index; NT-proBNP, N-terminal pro-B-type natriuretic peptide; NYHA, New York Heart Association; RVEDVi, right ventricular end-diastolic volume index; RVEF, right ventricular ejection fraction; VUS, variant of unknown significance

As for CMR findings, median LVEDVi was 94 mL/m^2^ (78–118), median LVEF was 51% (41–60), 76% had LGE in the LV, and 42% displayed fatty replacement in the LV. Furthermore, 2% displayed moderate-to-severe RV dilation according to the European Association of Cardiovascular Imaging consensus criteria [8]; 41% had RV wall motion abnormalities, 12% LGE and 32% fatty replacement in the RV. Fifty-four patients (14%) met the criteria for definite biventricular ACM. No patient had a defibrillator implanted at baseline.

### LVEDVi cut-off to define NDLVC

Over the 4.3-year median follow-up (1.9-7.0), a defibrillator was implanted in 140 patients (36%). Two patients (0.5%) received a cardiac resynchronization therapy device. The primary endpoint event occurred in 59 patients (15%), with 19 deaths, 40 VT and 19 VF episodes. All VT and VF episodes were terminated by defibrillator interventions. The secondary endpoint occurred in 57 patients, following 17 cardiac deaths and the same numbers of VT and VF episodes.

**Fig. 1:**
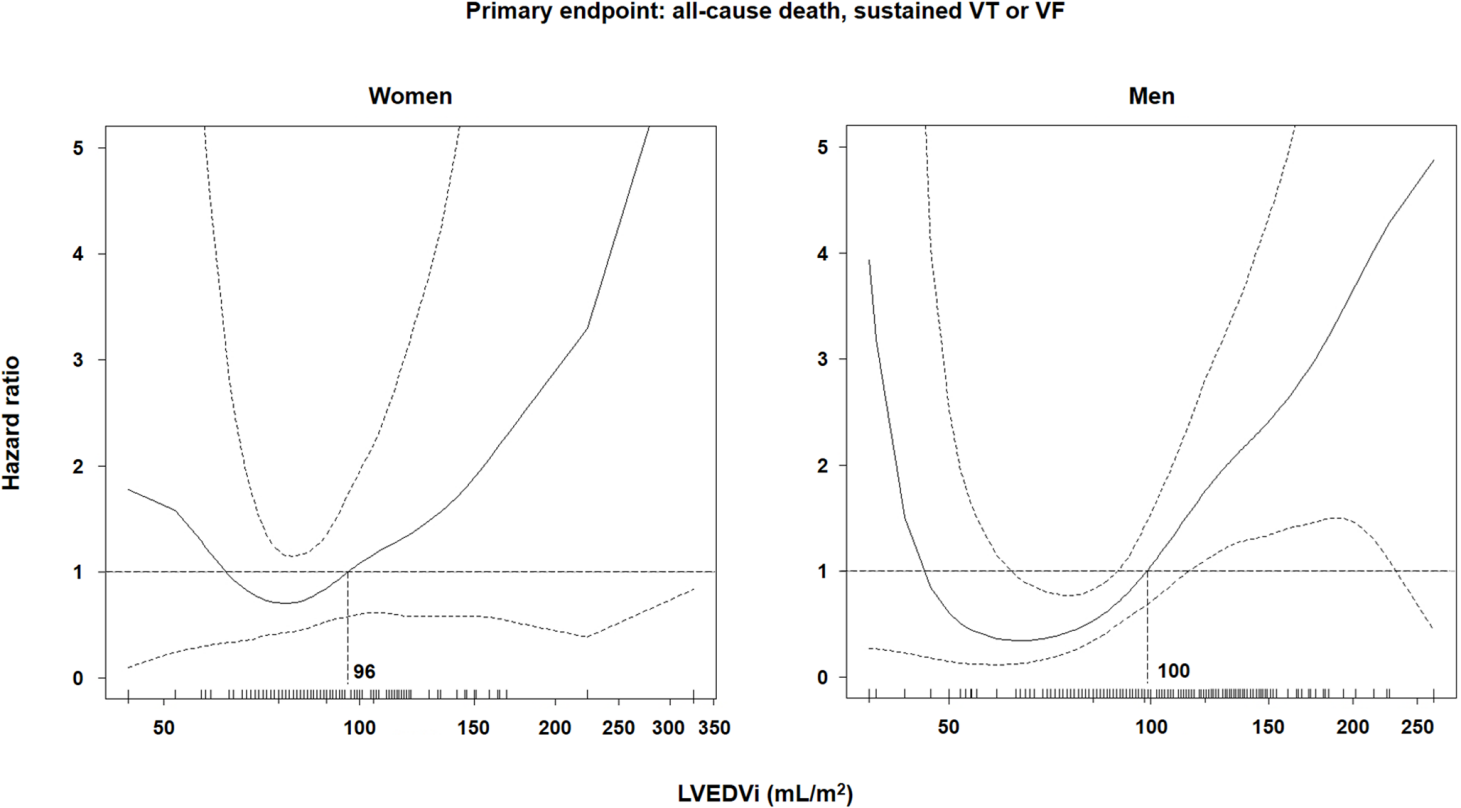
Risk of death or ventricular arrhythmias according to left ventricular end-diastolic volume index (LVEDVi). The curves of male and female patients are reported. The inflection points of the curves are provided. VF, ventricular fibrillation; VT, ventricular tachycardia

The risk of both endpoints increased exponentially with LVEDVi values (Fig. 1 and Supplemental Fig. 2). The inflection points of the curve for the primary endpoint were 96 mL/m^2^ in women and 100 mL/m^2^ in men, and the inflection points of the curve for the secondary endpoint were 100 mL/m^2^ in women and 93 mL/m^2^ in men. These values approached the upper reference limit of LVEDVi values (< 96 mL/m^2^ in women, < 105 mL/m^2^ in men) [2]. Accordingly, we defined NDLVC as LVEF < 55% and/or LV scarring with a non-ischemic pattern and/or fatty replacement, and LVEDVi values < 96 mL/m^2^ in women or < 105 mL/m^2^ in men.

Compared with patients with LV dilation, those with NDLVC had higher LVEF and lower LV mass index (both *p* < 0.001) as well as a lower number of LV segments with LGE (*p* = 0.033), but more often fatty replacement in the LV (*p* < 0.001). They had also a less dilated RV, but more often LGE and fatty replacement in the RV (*p* = 0.023 and *p* < 0.001, respectively; Table 2).

**Table 2:** Patients with non-dilated left ventricular cardiomyopathy (NDLVC) vs. those with LV dilation

	NDLVC (< 96 mL/m^2^ W, < 105 mL/m^2^ M) *n* = 237 (61%)	LV dilation (≥ 96 mL/m^2^ W, ≥ 105 mL/m^2^ M) *n* = 151 (39%)	*p*
Age (years)	56 (44–64)	54 (42–61)	0.136
Female sex, n (%)	81 (34)	44 (29)	0.300
Family history of SCD, n (%)	81 (35)	36 (25)	**0.036**
NT-proBNP (ng/L)	85 (41–207)	376 (152-1,072)	**< 0.001**
NYHA class I/II, n (%)	127/110 (54/46)	20/131 (13/87)	**< 0.001**
Hypertension, n (%)	68 (29)	25 (17)	0.067
Diabetes, n (%)	23 (10)	8 (5)	0.045
*Gene testing*			
Negative/VUS/LP or P variant, n (%)	131/51/55 (55/22/23)	94/21/36 (62/14/24)	0.160
LP or P variant in a desmosomal gene, n (%)	31 (13)	8 (5)	**0.013**
*CMR findings*			
LVEDVi (mL/m^2^)	82 (72–91)	123 (113–146)	**< 0.001**
LVEF (%)	56 (50–64)	40 (30–50)	**< 0.001**
LVMI (g/m^2^)	63 (55–73)	81 (68–94)	**< 0.001**
LV wall motion abnormalities, n (%)	89 (38)	129 (85)	**< 0.001**
LGE presence in the LV, n (%)	185 (78)	109 (72)	0.163
n of LV segments with LGE, n	2 (1–4)	3 (0–6)	**0.033**
Percent LGE mass (% of LV mass)	4 (0–9)	5 (0–14)	0.198
Fatty replacement in the LV, n (%)	125 (53)	37 (25)	**< 0.001**
n of LV segments with fatty replacement, n	1 (0–2)	0 (0–2)	0.187
RVEDVi (mL/m^2^)	78 (67–91)	88 (72–104)	**< 0.001**
Moderate-to-severe RV dilation, n (%)	0 (0)	6 (4)	**0.002**
RV wall motion abnormalities, n (%)	113 (48)	47 (31)	**< 0.001**
RVEF (%)	57 (52–63)	57 (46–62)	**0.048**
RV LGE, n (%)	35 (15)	11 (7)	**0.023**
Fatty replacement in the RV, n (%)	108 (46)	15 (10)	**< 0.001**
Definite biventricular ACM, n (%)	31 (13)	23 (15)	0.551

Percentages were calculated out of patients with available data. ACM, arrhythmogenic cardiomyopathy; CMR, cardiovascular magnetic resonance; LGE, late gadolinium enhancement; LP/P, likely pathogenic or pathogenic; LVEDVi, left ventricular end-diastolic volume index; LVEF, left ventricular ejection fraction; LVMI, left ventricular mass index;; NT-proBNP, N-terminal pro-B-type natriuretic peptide; NYHA, New York Heart Association; RVEDVi, right ventricular end-diastolic volume index; RVEF, right ventricular ejection fraction; VUS, variant of unknown significance

We then compared patients with NDLVC vs. patients with DCM, stratified according to a LVEF > 45% vs. ≤45% (Supplemental Table 2) or to a LVEF > 40% vs. ≤40% (Supplemental Table 3). Patients with NDLVC and preserved LVEF were older, but had no more often LGE in the LV or LP/P variants than those with DCM and preserved LVEF.

### NDLVC: preserved versus reduced LVEF

**Fig. 2:**
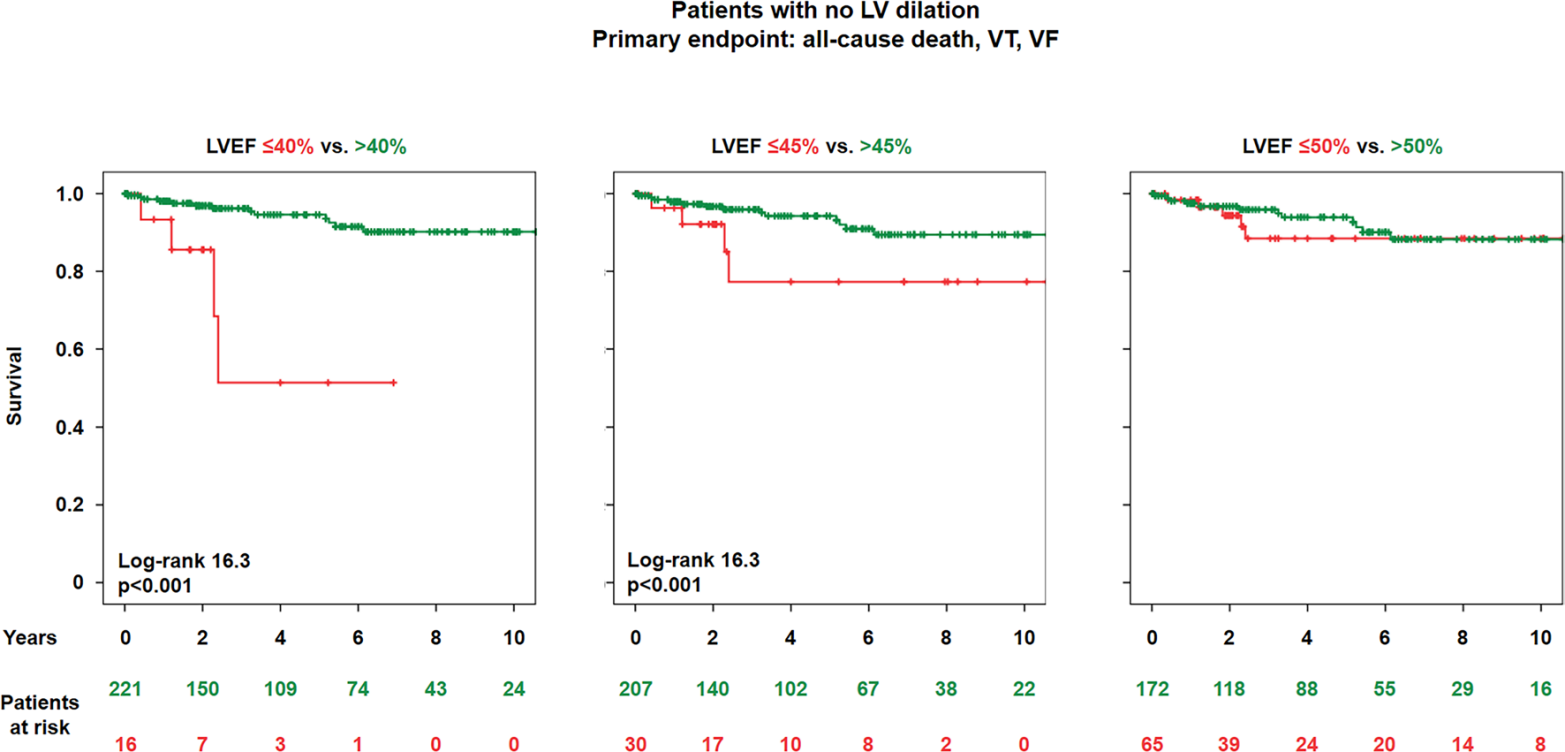
Patients with no left ventricular (LV) dilation: LV ejection fraction (LVEF) cut-offs and primary endpoint events. Kaplan-Meier survival curves. LVEDVi, LV end-diastolic volume index; M, men; W, women

We then searched for risk predictors within the NDLVC cohort. Among all baseline variables, only LVEF emerged as a univariate predictor of outcome, whereas fibro-fatty replacement, RV involvement or LP/P gene variants did not (Supplemental Table 4). LVEF was also the only univariable predictor of the secondary endpoint (hazard ratio 0.94, 95% confidence interval 0.89–0.99, *p* = 0.013). When considering possible LVEF thresholds for higher risk, we found that patients with LVEF ≤ 40% (*n* = 14) or LVEF ≤ 45% (*n* = 25) had a shorter survival than patients with LVEF > 40% or > 45%, respectively, while those with LVEF < 50% had not a shorter survival than those with LVEF > 50% (Fig. 2 and Supplemental Fig. 3).

Patients with NDLVC and LVEF either < 40% or < 45% were older, had more LV segments with LGE, as well as a less dilated RV, less common RV wall motion abnormalities and less fatty replacement in the RV than LNDLVC patients with preserved LV systolic function (Table 3).

**Table 3:** Patients with non-dilated left ventricular cardiomyopathy (NDLVC) and reduced vs. preserved LV ejection fraction

	LVEF ≤ 40% *n* = 16	LVEF > 40% *n* = 221	*p*	LVEF ≤ 45% *n* = 30	LVEF > 45% *n* = 207	*p*
Age (years)	63 (49–74)	55 (44–64)	**0.028**	64 (53–75)	54 (43–63)	**< 0.001**
Female sex, n (%)	3 (19)	78 (35)	0.178	10 (33)	71 (34)	0.917
Family history of SCD, n (%)	4 (25)	77 (35)	0.389	11 (37)	70 (34)	0.716
NT-proBNP (ng/L)	662 (289-1,598)	78 (38–168)	**< 0.001**	292 (158–676)	72 (35–143)	**< 0.001**
NYHA class I/II, n (%)	10/6 (63/37)	117/104 (53/47)	0.075	18/12 (60/40)	109/98 (53/47)	0.085
Hypertension, n (%)	4 (25)	64 (29)	0.288	7 (23)	61 (29)	0.355
Diabetes, n (%)	2 (13)	21 (10)	0.119	4 (13)	19 (9)	0.596
*Gene testing*						
Negative/VUS/LP or P variant, n (%)	10/3/3 (63/19/19)	121/48/52 (55/22/24)	0.831	17/5/8 (57/17/27)	114/46/47 (55/22/23)	0.754
LP or P variant in a desmosomal gene, n (%)	2 (13)	29 (13)	0.943	3 (10)	28 (14)	0.592
*CMR findings*						
LVEDVi (mL/m^2^)	94 (85–98)	82 (72–90)	**0.001**	91 (77–97)	81 (72–90)	**0.006**
LVEF (%)	36 (31–39)	57 (51–64)	**< 0.001**	40 (35–44)	59 (52–64)	**< 0.001**
LVMI (g/m^2^)	77 (64–90)	62 (55–72)	**< 0.001**	71 (56–84)	62 (55–72)	**0.047**
LV wall motion abnormalities, n (%)	15 (94)	74 (34)	**< 0.001**	29 (97)	60 (29)	**< 0.001**
LGE presence in the LV, n (%)	15 (94)	170 (77)	0.122	27 (90)	158 (76)	0.098
n of LV segments with LGE, n	4 (2–11)	2 (1–4)	**0.003**	3 (1–7)	2 (1–4)	**0.011**
Percent LGE mass (% of LV mass)	6 (3–19)	4 (0–9)	0.064	5 (3–15)	4 (0–8)	**0.044**
Fatty replacement in the LV, n (%)	6 (38)	119 (54)	0.193	11 (37)	114 (55)	0.052
n of LV segments with fatty replacement, n	3 (1–10)	1 (0–2)	0.124	3 (2–5)	1 (0–2)	**0.009**
RVEDVi (mL/m^2^)	57 (54–69)	79 (68–92)	**< 0.001**	65 (57–69)	80 (69–92)	**< 0.001**
Moderate-to-severe RV dilation, n (%)	0 (0)	0 (0)	**–**	0 (0)	0 (0)	**-**
RV wall motion abnormalities, n (%)	2 (13)	111 (50)	**0.004**	5 (17)	108 (52)	**< 0.001**
RVEF (%)	54 (47–58)	58 (52–64)	**0.007**	54 (48–57)	58 (52–64)	**0.001**
RV LGE, n (%)	2 (13)	33 (15)	0.770	3 (10)	32 (16)	0.410
Fatty replacement in the RV, n (%)	2 (13)	106 (48)	**0.005**	3 (10)	105 (51)	**< 0.001**
Definite biventricular ACM, n (%)	3 (19)	28 (13)	0.486	4 (13)	27 (13)	0.965

Percentages were calculated out of available values. Significant p values are reported in bold. CMR, cardiovascular magnetic resonance; LGE, late gadolinium enhancement; LP/P, likely pathogenic or pathogenic; LVEDVi, left ventricular end-diastolic volume index; LVEF, left ventricular ejection fraction; LVMI, left ventricular mass index; RVEDVi, right ventricular end-diastolic volume index; RVEF, right ventricular ejection fraction; VUS, variant of unknown significance

**Fig. 3:**
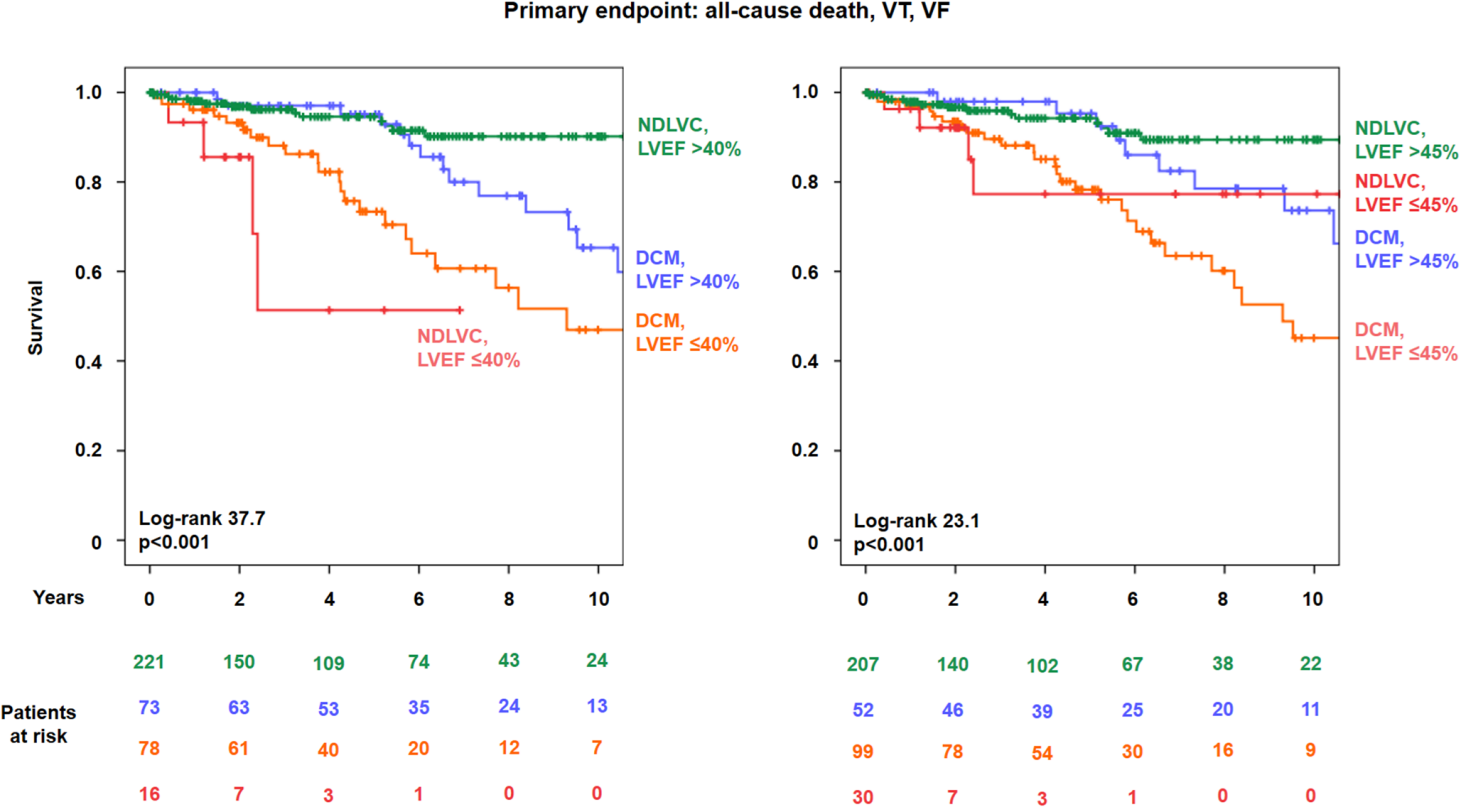
Survival according to left ventricular (LV) dilation and LV ejection fraction (LVEF) cut-offs: primary endpoint. DCM, dilated cardiomyopathy; NDLVC, non-dilated left ventricular cardiomyopathy

We then compared the survival of patients categorized according to LV dilation (NDLVC vs. DCM) and LVEF (> 45% or > 40% vs. ≤45% or ≤ 40%). Patients with NDLVC and LVEF ≥ 45% had the longest survival, and those with DCM and LVEF ≤ 45% the shortest, but very close to patients with NDLVC and LVEF ≤ 45% (Fig. 3). Patients with NDLVC and LVEF > 45% had a longer survival than those with NDLVC and LVEF ≤ 45% (*p* = 0.025, log-rank 5.0), and those with NDLVC and LVEF ≤ 45% had not a better prognosis than those with DCM and LVEF ≤ 45% (*p* = 0.690). When categorizing patients based on the LVEF 40% cut-point, patients with NDLVC and LVEF > 40% had the longest survival, and those with NDLVC and LVEF < 40% the shortest (even shorter than patients with DCM and LVEF ≤ 40%; Fig. 3). Similar findings emerged when consider the secondary endpoint (Supplemental Fig. 4).

## Discussion

**Fig. 4:**
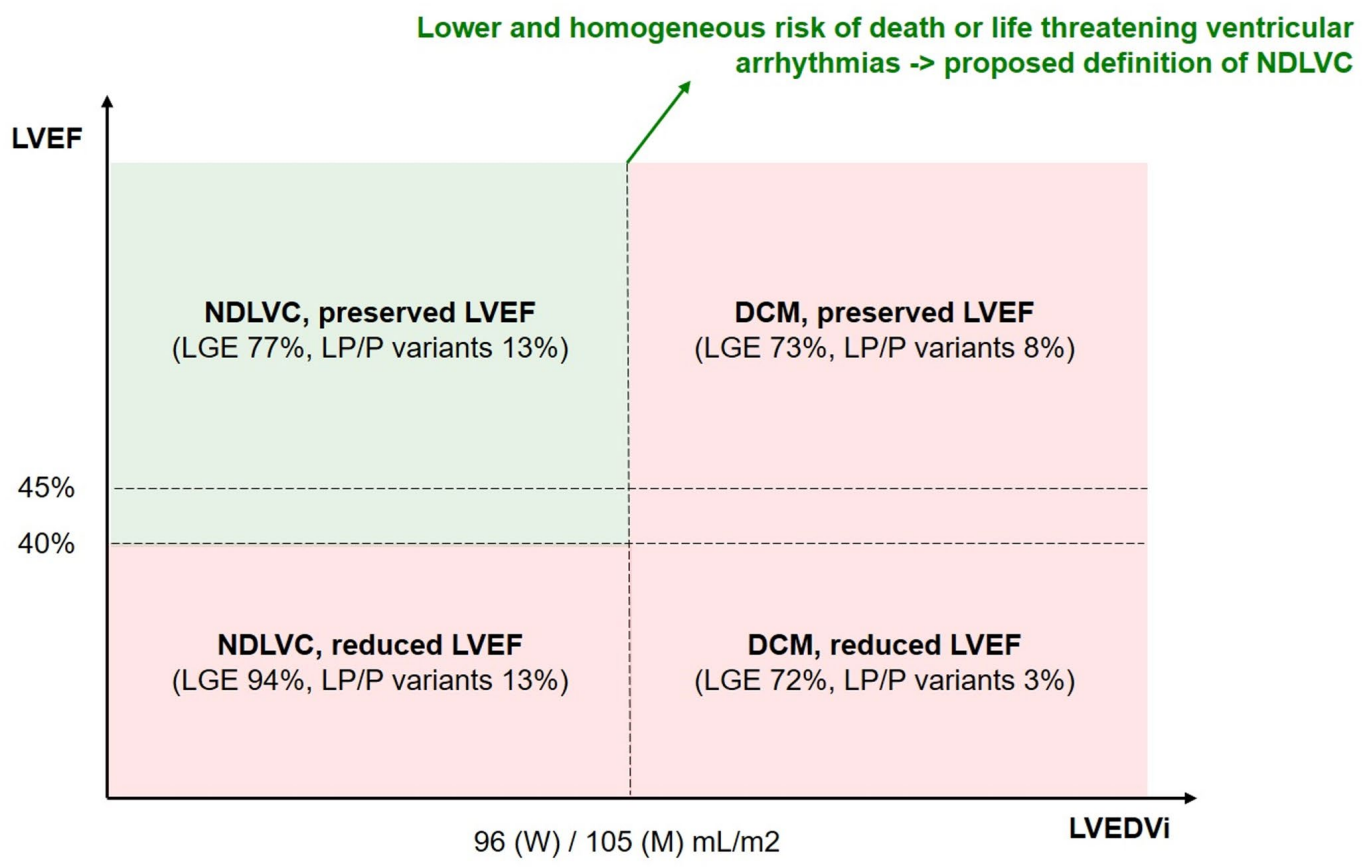
Central Illustration. Proposed diagnostic criteria for non-dilated left ventricular cardiomyopathy (NDLVC). See text for details. LGE, late gadolinium enhancement; LVEDVi, left ventricular end-diastolic volume index; LVEF, left ventricular ejection fraction; M, men; W, women

In this study we propose for the first time diagnostic thresholds that refine the diagnosis of NDLVC and identify a group of patients with a similar outcome. In a cohort of patients with nonischaemic cardiomyopathy, LV systolic dysfunction and/or LV scarring with a non-ischemic pattern and/or fatty replacement, there was an exponential relationship between LVEDVi values and the primary endpoint of all-cause death, sustained VT or VF, as well as the secondary endpoint of cardiac death, sustained VT or VF. Based on the inflection curves of the spline curves, we identified the sex-specific upper reference values of LVEDVi as the thresholds identifying a patient group with a homogeneous prognosis. Among these patients, LVEF was a strong predictor of outcome, with an increased risk for values ≤ 45% and particularly ≤ 40% (i.e., the current threshold for heart failure with reduced EF) [12]. Therefore, we suggest incorporating these criteria for LV involvement (LVEDVi < 96 mL/m^2^ in women or < 105 mL/m^2^ in men plus LVEF ≥ 40% or ≥ 45%) in the definition of NDLVC to enhance the prognostic value of the NDLVC classification (Central Illustration-Fig. 4).

Interestingly, the presence and extent of LGE and fatty replacement in the LV were not predictive of outcome in NDLVC, possibly because they are inclusion criteria. Indeed, the unexpected lack of prognostic value of LGE is reasonably explained by its high prevalence in patients without LV dilation (78%). In a study on early non-ischemic cardiomyopathy patients, myocardial LGE was an independent predictor of cardiovascular events, but its prevalence was much lower (26%) than our study, likely because the study included only patients with either LV dilation and/or dysfunction, not patients with isolated fibro-fatty replacement as per NDLVC definition [13]. In another multicenter study on DCM/NDLVC [14], septal LGE resulted a significant prognostic predictor of arrhythmic events, but overall LGE prevalence (60%) was still significantly lower than our study, likely because of the relatively higher proportion of patients with LV dilatation/systolic dysfunction, compared to patients with isolated fibro-fatty replacement. Similarly, in a recent sub-analysis of the DERIVATE study on 197 NDLVC patients, LGE was present in 43% of patients and resulted an independent predictor of arrhythmic events [15].

A striking (and previously under-emphasized) observation is the high prevalence of RV abnormalities that are classically associated with ARVC within the NDLVC cohort: 41% showed RV wall-motion abnormalities, 32% fatty replacement and 12% RV LGE. On the other hand, only 14% of patients presented with a definite arrhythmogenic biventricular cardiomyopathy according to the recently published European Task Force criteria [8], while no patient presented with isolated ARVC, which was one of the exclusion criteria. Although RV variables were not independently prognostic after restricting the analysis to patients without LV dilation, their frequency suggests the existence of an arrhythmogenic, fibro-fatty phenotype that spans both ventricles and may represent a distinct pathophysiological subset of NDLVC. Several mechanisms may underlie this overlap. First, desmosomal variants (which accounted for 10% of likely pathogenic mutations in our population) predispose to bi-ventricular or left-dominant arrhythmogenic cardiomyopathy and could foster fibro-fatty replacement in both chambers, even before dilation ensues. Second, shared molecular pathways (e.g., Wnt/β-catenin signaling suppression) [16] might promote fatty metaplasia irrespective of ventricular size. Finally, early involvement of the RV could modulate LV remodeling through interventricular interactions or by acting as an arrhythmogenic trigger. Clinical corollaries are two-fold. First, when NDLVC is accompanied by right ventricular functional or structural abnormalities, clinicians should consider the diagnosis of arrhythmogenic biventricular cardiomyopathy. Second, the lack of additional prognostic weight of RV findings in the present study does not preclude their importance. While the partial overlap between NDLVC and arrhythmogenic LV cardiomyopathy has been investigated [17], further multicenter studies with systematic biventricular characterization are required to investigate the boundaries between NDLVC and ARVC.

At first sight, the 61% prevalence of non-dilated ventricles appears striking; however, three mechanistic considerations explain this enrichment. Our inclusion algorithm deliberately excluded even mild LV enlargement, applying healthy-reference LVEDVi thresholds up-front. Furthermore, the study was carried out in a tertiary referral center, where patients are frequently referred because of ventricular arrhythmias, gene-positive status or worrying family histories, before overt structural remodeling occurs. Finally, several arrhythmogenic genes promote cell-cell uncoupling, apoptosis and adipogenesis; focal fibro-fatty scars may therefore precede eccentric remodeling for years, generating the “concealed” arrhythmic phase captured by contemporary CMR protocols.

A pathogenic or likely-pathogenic (LP/P) variant was present in 24% of patients, with desmosomal mutations accounting for 10%, figures appreciably higher than in unselected DCM registries [18]. Desmosomal (*PKP2*,* DSG2*), lamin (*LMNA*) and truncating *FLNC* variants destabilize the cytoskeleton, activate inflammatory and adipogenic programmes and shorten action-potential duration, changes that favor ventricular arrhythmias long before global LV dilatation. This genetic architecture therefore provides a biological rationale for the twin observations of frequent non-dilated ventricles and high arrhythmic burden despite preserved chamber size.

A sizeable minority of index cases were investigated because of a family history of sudden cardiac death (SCD; Table 1). Familial clustering is expected in desmosomal or LMNA-mediated disease, in which malignant ventricular arrhythmias may occur at the “concealed” or inflammatory hot-phase stage, again preceding dilatation. Synergistic mechanisms include: genetic arrhythmogenic substrate (fibro-fatty scars, ion-channel remodeling); adrenergic triggers during daily life; electrical inhomogeneity introduced by patchy scar tissue. The over-representation of families with SCD therefore fits the molecular profile of our cohort and reinforces the need for cascade screening in relatives.

Our findings have clear implications for clinical practice, suggesting a more refined framework for diagnosing and managing patients with suspected NDLVC. Incorporating LVEDVi and LVEF thresholds into the ESC definition of NDLVC allows clinicians to separate truly early/arrhythmogenic phenotypes from latent DCM, facilitating tailored surveillance. Additionally, the conjunction of frequent pathogenic variants and familial SCD highlights the importance of systematic cascade testing, early CMR and lifestyle counselling in relatives.

Our primary aim was to implement the NDLVC definition by proposing evidencebased cutoffs that confirm the absence of LV dilation and delineate LVEF ranges that cluster patients into clinically homogeneous groups. Conceptually, establishing clear boundaries of the NDLVC spectrum is a prerequisite to characterizing its phenotypic subsets, and larger, multicenter studies are now needed to validate these proposed criteria and confirm their applicability in routine practice.

Several limitations should be acknowledged in this study. First, this was a single-center study with a retrospective design. The exclusion of patients without available genetic data (resulting in a final cohort of 388 out of 1,250 initially eligible individuals) was necessary to ensure consistent and complete phenotypic and genotypic characterization. While this introduces a risk of selection bias and limits the generalizability of our findings to unselected populations, it reflects the diagnostic standard of care at our tertiary center, where full cardiomyopathy workup routinely includes genetic testing. This approach enabled rigorous internal comparisons but may have enriched the cohort for more complex cases, including those with a higher prevalence of adverse events and imaging abnormalities such as LGE. In our analysis we also excluded specific cardiac diseases, as myocarditis, sarcoidosis, amyloidosis and other inflammatory or storage disease should be considered separately from conventional DCM or NDLVC, also because their prognostic and therapeutic management are highly dependent on specific etiologies. Second, the number of patients and events was quite limited. For example, survival analysis for patients with NDLVC and LVEF ≤ 40% should be interpreted with caution due to the small sample size (*n* = 16). Even the follow-up duration was quite short, limiting the possibility to investigate the development of heart failure. Third, the prognostic value of fibrous and/or fatty replacement in NDLVC are complicated by their high prevalence as inclusion criteria, as well as by their high heterogeneity. Larger studied are needed to investigate the prognostic role of more specific LGE and fatty patterns, locations or extent, because their presence alone cannot identify high-risk patients. Several previous studies have investigated the prognostic significance of LGE patterns, locations and extent [7, 19], of native T1 [20, 21] and extracellular volume mapping [22, 23] in DCM patients, and similar analysis might potentially provide prognostically useful data also in NDLVC patients.

In conclusion, patients with NDLVC, identified by the presence of non-ischemic LV scarring and/or fatty replacement and/or hypokinesia and LVEDVi < 96 mL/m^2^ (women) or < 105 mL/m^2^ (men), have a lower risk of death or ventricular arrhythmias compared to DCM patients. Among these patients, those with LVEF ≥ 45% or ≥ 40% have a much lower risk of events, but NDLVC patients with a LVEF < 45% or < 40% presented a much worse prognosis. The definition of NDLVC could incorporate these LVEDVi and LVEF cut-points to identify a population of patients with a homogeneous risk of death or ventricular arrhythmias.

### Clinical competencies

The study offers insights into non-dilated left ventricular cardiomyopathy (NDLVC) by refining diagnostic criteria and linking these to patient outcomes. This work provides clinicians with a more precise approach to identifying NDLVC patients who may be at a lower risk of adverse events compared to those with dilated cardiomyopathy (DCM). By implementing these findings, healthcare providers can more accurately stratify patients based on risk and guide the clinical decision-making process for monitoring and treating NDLVC, particularly using left ventricular ejection fraction (LVEF) and left ventricular end-diastolic volume index (LVEDVi) as key parameters. This improves the clinician’s ability to deliver patient-specific care.

### Translational outlook

The study’s definition of NDLVC, incorporating LVEDVi and LVEF thresholds, sets the stage for future research into the molecular and genetic underpinnings of non-dilated cardiomyopathy. Additional research is needed to understand the prognostic value of myocardial scarring and fatty replacement and their relationship to genetic markers. Future investigations should focus on expanding these findings across broader populations and integrating new imaging technologies for more precise characterization of NDLVC. Furthermore, the development of personalized treatment strategies based on genetic profiling and advanced imaging could enhance the prognosis and management of NDLVC, advancing towards a more individualized approach to cardiomyopathy care.

## Supplementary Information

Below is the link to the electronic supplementary material.

Supplementary Material 1

## Data Availability

Data will be made available upon request to the corresponding author.

## Abbreviations

ARVC: Arrhythmogenic right ventricular cardiomyopathy
CMR: Cardiovascular magnetic resonance
DCM: Dilated cardiomyopathy
LGE: Late gadolinium enhancement
LP/P: Likely pathogenic or pathogenic
LV(EF): Left ventricular (ejection fraction)
LVEDVi: Left ventricular end-diastolic volume index
NDLVC: Non-dilated left ventricular cardiomyopathy
RV: Right ventricular
VF/VT: Ventricular fibrillation/tachycardia
