# Supplementary material for: A prognostically meaningful definition of non-dilated left ventricular cardiomyopathy
Source: Int J Cardiovasc Imaging. 2025 Jul 31;41(9):1749–58. doi: 10.1007/s10554-025-03474-y (PMC12405037; doi:10.1007/s10554-025-03474-y)
Supplement: Supplementary file 1 — Supplementary Material 1 [file 10554_2025_3474_MOESM1_ESM.docx]

**Supplemental material**

**A prognostically meaningful definition**

**of non-dilated left ventricular cardiomyopathy**

Alberto Aimo^1,2^, Ignazio Alessio Gueli^1,2^, Bianca Alderotti^3^, Irina Bellisario^4^, Giancarlo Todiere^2^, Chrysanthos Grigoratos^2^, Carmelo De Gori^5^, Alberto Clemente^5^, Giorgia Panichella^6^, Giuseppe Vergaro^1,2^, Alberto Giannoni^1,2^, Nicoletta Botto^2^, Simona Vittorini^2^, Claudio Passino^1,2^, Giovanni Donato Aquaro^7^, Filippo Cademartiri^5^, Michele Emdin^1,2^, Andrea Barison^1,2^

1. Interdisciplinary Center for Health Sciences, Scuola Superiore Sant’Anna, Pisa, Italy; 2. Fondazione Toscana Gabriele Monasterio, Pisa and Massa, Italy. 3. Emergency Medicine, Azienda ospedaliera Universitaria Pisana; 4. Department of Neuroscience, Imaging and Clinical Sciences, University of Chieti; 5. Radiology Department, Fondazione Toscana Gabriele Monasterio, Pisa; 6. Cardiology Division, Careggi University Hospital, Florence, Italy; 7. Academic Radiology Unit, Department of Surgical, Medical and Molecular Pathology and Critical Area, University of Pisa.

**Supplemental Table 1. Gene variants found in the cohort.**

| **LVEF** | **LV not dilated** | **Gene 1** | **Variant** | **Classification** | **Gene 2** | **Variant** | **Classification** | **Gene 3** | **Variant** | **Classification** |
| --- | --- | --- | --- | --- | --- | --- | --- | --- | --- | --- |
| 79 | 1 | *DSG2* | c. 1912 G>A | P |  |  |  |  |  |  |
| 77 | 1 | *TMEM* | c.1150 C>G | P |  |  |  |  |  |  |
| 76 | 1 | *MYH7* | c.4816 C>T | VUS |  |  |  |  |  |  |
| 75 | 1 | *MYBPC3* | c.2870 C>G | VUS |  |  |  |  |  |  |
| 75 | 1 | *TMEM* | c.257 G>A | VUS |  |  |  |  |  |  |
| 73 | 1 | *DSG2* | c.473 T>G | VUS |  |  |  |  |  |  |
| 72 | 1 | *RYR2* |  | VUS | *JUP* |  | VUS |  |  |  |
| 71 | 1 | *DSP* | c.3922 C>T | VUS (likely benign) |  |  |  |  |  |  |
| 71 | 1 | *PKP2* | c.505 A>G | VUS |  |  |  |  |  |  |
| 70 | 1 | *DSC2* | c.1249 delG | VUS | *TRPM4* | c.857 G>T | VUS |  |  |  |
| 69 | 1 | *MYH7* |  |  | *SCN5A* |  | VUS |  |  |  |
| 69 | 1 | *SCN5A* | c.5336 C>T | LP VUS |  |  |  |  |  |  |
| 68 | 1 | *DSG2* | c.727 G>T | P |  |  |  |  |  |  |
| 67 | 1 | *DSC2* | c.836 G>A | P |  |  |  |  |  |  |
| 65 | 1 | *DSC2* | c.901 A>G | VUS |  |  |  |  |  |  |
| 65 | 1 | *DSP* | c.3922 C>T | VUS | *KCNE1* | c.13432 T>C | VUS |  |  |  |
| 65 | 1 | *MYH7* | c.3865 C>T | VUS | *EYA4* | c.1411 G>A | VUS |  |  |  |
| 65 | 1 | *PKP2* | c.2443_2448 delAACACC/insGAAA | P |  |  |  |  |  |  |
| 64 | 1 | *ABCC9* | c.2504 T>C | LP VUS |  |  |  |  |  |  |
| 64 | 1 | *DSP* | c. 88 G>A | VUS (likely benign) |  |  |  |  |  |  |
| 64 | 1 | *JUP* | c.607 C>T | VUS |  |  |  |  |  |  |
| 64 | 1 | *MYH7* | c.253 G>A | VUS | *SCN5A* | c.1705 C>T | VUS |  |  |  |
| 64 | 1 | *PKP2* |  | VUS |  |  |  |  |  |  |
| 63 | 1 | *DSP* | c.2723 G>A | VUS (likely benign) |  |  |  |  |  |  |
| 63 | 1 | *DSP* | c.7207 delG | P |  |  |  |  |  |  |
| 63 | 1 | *JUP* | c.412 G>A | VUS |  |  |  |  |  |  |
| 63 | 1 | *PKP2* | c.1759 G>A | VUS |  |  |  |  |  |  |
| 62 | 1 | *DSG2* | c.2759 T>G | VUS |  |  |  |  |  |  |
| 62 | 1 | *DSG2* | c.428 T>A | P |  |  |  |  |  |  |
| 62 | 1 | *DSP* | c.88 G>A | P |  |  |  |  |  |  |
| 62 | 1 | *DSP* | c.88 G>A | P |  |  |  |  |  |  |
| 62 | 1 | *PKP2* | c.1063 C>T c.1583 C>G | P |  |  |  |  |  |  |
| 62 | 1 | *PKP2* | c.2443_2448 delAACACC/insGAAA | P |  |  |  |  |  |  |
| 61 | 1 | *ABCC9* |  | VUS |  |  |  |  |  |  |
| 61 | 1 | *DSP* | c.2183 A>G | VUS |  |  |  |  |  |  |
| 61 | 1 | *DSP* | c.5249 T>G | VUS |  |  |  |  |  |  |
| 60 | 1 | *DSP* | c.166 G>A | P |  |  |  |  |  |  |
| 60 | 1 | *DSP* | c.88 G>A | P |  |  |  |  |  |  |
| 60 | 1 | *DSP* | c.2471 T>A | P |  |  |  |  |  |  |
| 60 | 1 | *PRDM16* | c.478 G>A | VUS | *ANK2* | c.11716 C>T | VUS |  |  |  |
| 59 | 1 | *DES* | c.170 C>T | VUS |  |  |  |  |  |  |
| 59 | 1 | *DSP* | c.6269 A>G | P |  |  |  |  |  |  |
| 59 | 1 | *PKP2* | c.2013delC | P |  |  |  |  |  |  |
| 58 | 1 | *DSG2* | c.1936 C>G | VUS |  |  |  |  |  |  |
| 58 | 1 | *DSP* | c.2759 T>G | VUS | *DSG2* | c.4105 G>A | VUS |  |  |  |
| 58 | 1 | *TTN* | c.102509 G>A | LP VUS |  |  |  |  |  |  |
| 57 | 1 | *CACNA1C* | c.5086 G>A | VUS |  |  |  |  |  |  |
| 57 | 1 | *DES* | c.634 C>T | P |  |  |  |  |  |  |
| 57 | 1 | *DSP* | c.5218 G>A | VUS |  |  |  |  |  |  |
| 57 | 1 | *DSP* | c.88 G>A | P |  |  |  |  |  |  |
| 57 | 1 | *MYH7* | c.4040 A>G | VUS | *RYR2* | c.3619 C>G | VUS |  |  |  |
| 56 | 1 | *DSG2* | c.166 G>A | VUS |  |  |  |  |  |  |
| 56 | 1 | *DSG2* | c.1003 A>G | P |  |  |  |  |  |  |
| 55 | 1 | *DSC2* | c.235 G>A | VUS |  |  |  |  |  |  |
| 55 | 1 | *DSP* | c.2464 A>G | P |  |  |  |  |  |  |
| 54 | 1 | *DSC2* | c.1018 A>G | VUS |  |  |  |  |  |  |
| 54 | 1 | *DSG2* | c.428 T>A | P |  |  |  |  |  |  |
| 54 | 1 | *DSP* | c.3282 G>C | VUS | *MYBPC3* | c.2873 C>T | VUS | KCNH2 | c.391 G>T | VUS |
| 54 | 1 | *DSP* | c.88 G>A | P |  |  |  |  |  |  |
| 54 | 1 | *DSP* | c.4198 C>T | P |  |  |  |  |  |  |
| 54 | 1 | *DTNA* | c.620 A>G | VUS |  |  |  |  |  |  |
| 54 | 1 | *KCNA5* |  | VUS |  |  |  |  |  |  |
| 54 | 1 | *KCNJ2* | c.13 C>T | VUS | *RBM20* | c.2183_2185del | VUS |  |  |  |
| 54 | 1 | *KCNQ1* | c.728 C>T | VUS |  |  |  |  |  |  |
| 54 | 1 | *LMNA* | c.565 C>T | P |  |  |  |  |  |  |
| 54 | 1 | *PRKAG2* | c.1159 G>A | VUS |  |  |  |  |  |  |
| 53 | 1 | *DSC2* | c.1668 dupG | P |  |  |  |  |  |  |
| 53 | 1 | *LMNA* | c.568 C>T | P |  |  |  |  |  |  |
| 53 | 1 | *LMNA* | c.565 C>T | P |  |  |  |  |  |  |
| 53 | 1 | *TNNI3* | c.479 G>A | P |  |  |  |  |  |  |
| 53 | 1 | *TTN* | c.91615_91621del | P |  |  |  |  |  |  |
| 52 | 1 | *LMNA* | c.1381 G>T | P |  |  |  |  |  |  |
| 52 | 1 | *PKP2* | c.1643 delG | P |  |  |  |  |  |  |
| 52 | 1 | *TTN* | c.68824 G>A | VUS |  |  |  |  |  |  |
| 51 | 1 | *DSP* | c. 1825 C>T | P |  |  |  |  |  |  |
| 51 | 1 | *DSP* | c.2821 C>T | P |  |  |  |  |  |  |
| 51 | 1 | *RYR2* | c.4841 G>A | VUS |  |  |  |  |  |  |
| 51 | 1 | *RYR2* | c.10837 A>G | LP VUS |  |  |  |  |  |  |
| 51 | 1 | *SCN3B* | c.29 T>C | VUS | *RYR2* | c.2755 G>A | VUS |  |  |  |
| 50 | 1 | *DSG2* | c.1003 A>G c.175 C>T | P |  |  |  |  |  |  |
| 50 | 1 | *MYH7* | c.732+1 G>T | P |  |  |  |  |  |  |
| 50 | 1 | *TNNI3* | c.428 C>A | VUS | *TNNT2* | c.482 G>A | VUS |  |  |  |
| 49 | 1 | *DSP* | c.7567_7570 delAAGA | P |  |  |  |  |  |  |
| 49 | 1 | *LDB3* | c.566 C>T | VUS |  |  |  |  |  |  |
| 49 | 1 | *TTN* | c.13335 delT | P |  |  |  |  |  |  |
| 48 | 1 | *DSP* | c.7567_7570 delAAGA | P |  |  |  |  |  |  |
| 48 | 1 | *EYA4* | c.878 A>G | VUS |  |  |  |  |  |  |
| 48 | 1 | *PLN* | c.40_42 del AGA | P |  |  |  |  |  |  |
| 48 | 1 | *SCN5A* | c.655 c>t | LP VUS |  |  |  |  |  |  |
| 48 | 1 | *TNNT2* | c.481 C>G | LP VUS |  |  |  |  |  |  |
| 47 | 1 | *LAMA4* | c.2828 c>t | VUS |  |  |  |  |  |  |
| 46 | 1 | *MYL3* | c.187C>T | VUS | *LDB3* |  | VUS | *NEXN* |  | VUS |
| 46 | 1 | *TTN* | c.46336_46339 dup | LP VUS |  |  |  |  |  |  |
| 45 | 1 | *KCNE3* | c.284 A>G | VUS |  |  |  |  |  |  |
| 45 | 1 | *TTN* | c.96407del C | P |  |  |  |  |  |  |
| 45 | 1 | *TTN* | c.104540_104541 insATAT | P |  |  |  |  |  |  |
| 43 | 1 | *DES* | c.1322 A>T | VUS |  |  |  |  |  |  |
| 43 | 1 | *DSP* | c.1825 C>T | P |  |  |  |  |  |  |
| 42 | 1 | *MYH7* | c.5717 C>G | P | *DSP* | c.8508_8519 dup | VUS |  |  |  |
| 42 | 1 | *TNNI3* | c.479 G>A | P |  |  |  |  |  |  |
| 40 | 1 | *TTN* | c.85011_85014 del | P |  |  |  |  |  |  |
| 39 | 1 | *SCN3B* | c.29 T>C | VUS |  |  |  |  |  |  |
| 38 | 1 | *SCN5A* | c.1844 G>A | VUS |  |  |  |  |  |  |
| 33 | 1 | *DSP* | c.4198 C>T | P | *TNNI3* | c.258 delC | P |  |  |  |
| 32 | 1 | *NEXN* | c.1579_1584 del | VUS | *RYR2* | c.13718 G>A | VUS |  |  |  |
| 19 | 1 | *DSP* | c.7207_7207delG | P |  |  |  |  |  |  |
| 66 | 0 | *PKP2* | c.1378+1 G>C | P |  |  |  |  |  |  |
| 62 | 0 | *KCNA5* |  | VUS | *MYH6* |  | VUS |  |  |  |
| 58 | 0 | *MYH7* | c.4884 G>T | VUS |  |  |  |  |  |  |
| 58 | 0 | *TNNI3* | c.220 C>T | VUS |  |  |  |  |  |  |
| 55 | 0 | *DSP* | c.3973 A>C | VUS |  |  |  |  |  |  |
| 54 | 0 | *LMNA* | c.350 A>G | P |  |  |  |  |  |  |
| 54 | 0 | *TTN* | c.65858_65861dupTGTT | P |  |  |  |  |  |  |
| 53 | 0 | *DES* | c.1009 G>A | VUS |  |  |  |  |  |  |
| 53 | 0 | *PKP2* | c.1504 C>T | P | *HCN4* | c.650 A>G | VUS |  |  |  |
| 53 | 0 | *SCN5A* | c.3388 G>A | VUS |  |  |  |  |  |  |
| 51 | 0 | *DES* | c.833 G>A | P |  |  |  |  |  |  |
| 51 | 0 | *DSP* | c.4483 A>G | VUS |  |  |  |  |  |  |
| 51 | 0 | *DSP* | c.2821 C>T | P |  |  |  |  |  |  |
| 51 | 0 | *TTN* | c.56806 C>T | P | *DSG2* | c.1003 A>G | VUS |  |  |  |
| 51 | 0 | *TTN* | c.105828 del A | LP VUS |  |  |  |  |  |  |
| 50 | 0 | *SCN5A* | c.1820 G>A | VUS | *MYPN* | c.845 A>G | VUS |  |  |  |
| 49 | 0 | *LMNA* | c.1381 G>T | P |  |  |  |  |  |  |
| 49 | 0 | *MYH7* | c.976 G>C | VUS | *JPH2* | c.1057 C>T | VUS |  |  |  |
| 49 | 0 | *SCN5A* | c.1844G>A | VUS |  |  |  |  |  |  |
| 47 | 0 | *DSP* |  | P |  |  |  |  |  |  |
| 47 | 0 | *RBM20* | c.2674 G>T | P | *LDB3* | c.566 C>T | VUS |  |  |  |
| 46 | 0 | *MYH6* | c.4471 G>A | P | *RYR2* | c.4585 A>C | VUS |  |  |  |
| 46 | 0 | *TTN* | c.29421 G>T | VUS |  |  |  |  |  |  |
| 45 | 0 | *DMD* | c.12G>A | P |  |  |  |  |  |  |
| 44 | 0 | *DSG2* | c.828_828+2 delGGT | P |  |  |  |  |  |  |
| 44 | 0 | *LMNA* | c.328 C>A | P |  |  |  |  |  |  |
| 44 | 0 | *TNNI3* | c.479 G>A | P |  |  |  |  |  |  |
| 44 | 0 | *TNNT2* | c.586 C>T | P |  |  |  |  |  |  |
| 43 | 0 | *MYH6* | c.2848 T>A | VUS |  |  |  |  |  |  |
| 43 | 0 | *MYH7* | c.5157 G>A | VUS |  |  |  |  |  |  |
| 42 | 0 | *DSP* | c.2848 delA | P |  |  |  |  |  |  |
| 41 | 0 | *DSP* | c.2431 C>A | P |  |  |  |  |  |  |
| 41 | 0 | *TTN* | c.12990del | LP VUS |  |  |  |  |  |  |
| 40 | 0 | *MYH6* |  | VUS | *MYH6* |  | VUS |  |  |  |
| 40 | 0 | *TTN* | c.96407del C | P |  |  |  |  |  |  |
| 39 | 0 | *MYH7* | c.2420 G>A | P |  |  |  |  |  |  |
| 38 | 0 | *LDB3* | c.566 C>T | VUS | *KCNJ2* | c.1151 G>A | VUS | MYPN | c.1475 C>T | VUS |
| 37 | 0 | *DES* | c.407 T>A | LP VUS | *TTN* | c.53182 g>t | LP VUS | SCN1B | c.536 g>a | LP VUS |
| 36 | 0 | *DSP* | c.5498 A>T | VUS | *DSG2* | c.907 G>A | VUS |  |  |  |
| 35 | 0 | *DSP* | c.7567_7570 delAAGA | P |  |  |  |  |  |  |
| 35 | 0 | *RBM20* | c.1907 G>A | P |  |  |  |  |  |  |
| 33 | 0 | *LMNA* | c.1189 C>T | VUS |  |  |  |  |  |  |
| 32 | 0 | *TTN* | c.69936 C>G | P |  |  |  |  |  |  |
| 31 | 0 | *DSP* | c.6328 G>C | homozigous |  |  |  |  |  |  |
| 30 | 0 | *RAF1* | c.464 G>A | P |  |  |  |  |  |  |
| 30 | 0 | *RYR2* | c.10282_10283 delAT | P |  |  |  |  |  |  |
| 29 | 0 | *DSG2* | c. 994 G>C | P |  |  |  |  |  |  |
| 29 | 0 | *LMNA* | c.1146 C>T | P |  |  |  |  |  |  |
| 27 | 0 | *RYR2* |  | VUS |  |  |  |  |  |  |
| 26 | 0 | *MYH7* | c.1766_1768 delACA | P |  |  |  |  |  |  |
| 26 | 0 | *TTN* | c.65858_65861dupTGTT | P |  |  |  |  |  |  |
| 23 | 0 | *RBM20* | c.3321_3328del | P | *BAG3* | c.394 C>T | P |  |  |  |
| 21 | 0 | *TTN* | c.88008_88009 delTG | P | *BAG3* | c.116 T>C | VUS |  |  |  |
| 19 | 0 | *DSP* | c.7207 delG | P | *MYH7* | c.5342 G>A | P |  |  |  |
| 17 | 0 | *MYH7* | c.5326 A>G | P |  |  |  |  |  |  |
| 16 | 0 | *LAMA4* | c.2471 A>G | LP VUS |  |  |  |  |  |  |
| 16 | 0 | *SCN5A* | 4748 G>A | VUS |  |  |  |  |  |  |

LVEF, left ventricular ejection fraction; LP, likely pathogenic; P, pathogenic; VUS, variant of unknown significance.

**Supplemental Table 2. Patient characteristics according to the left ventricular ejection fraction (LVEF) cut-off 45% and LV dilation.**

|  | **LVEF >45%**  **n=259** | | | **LVEF ≤45%**  **n=129** | | |
| --- | --- | --- | --- | --- | --- | --- |
|  | **NDLVC**  **n=207 (80%)** | **DCM**  **n=52 (20%)** | **p** | **NDLVC**  **n=30 (23%)** | **DCM**  **n=99 (77%)** | **p** |
| Age (years) | 54 (43-63) | 46 (37-59) | **0.011** | 64 (53-75) | 56 (47-63) | **0.001** |
| Female sex, n (%) | 71 (34) | 14 (27) | 0.311 | 10 (33) | 30 (30) | 0.753 |
| Family history of SCD, n (%) | 70 (34) | 13 (25) | 0.252 | 11 (37) | 23 (23) | 0.138 |
| NT-proBNP (ng/L) | 72 (35-143) | 185 (87-282) | **<0.001** | 292 (158-676) | 595 (215-1,328) | 0.078 |
| NYHA class I/II, n (%) | 109/98 (53/47) | 12/40 (23/77) | **<0.001** | 18/12 (60/40) | 8/91 (8/92) | **<0.001** |
| Hypertension, n (%) | 61 (29) | 13 (25) | 0.171 | 7 (23) | 12 (12) | **<0.001** |
| Diabetes, n (%) | 19 (9) | 5 (10) | 0.367 | 4 (13) | 3 (3) | **<0.001** |
| Gene testing | | | | | | |
| Negative/VUS/LP or P variant, n (%) | 114/46/47 (55/22/23) | 29/11/12 (56/21/23) | 0.986 | 17/5/8 (57/17/27) | 65/10/24 (66/10/24) | 0.549 |
| LP or P variant in a desmosomal gene, n (%) | 28 (14) | 3 (6) | 0.123 | 3 (10) | 5 (5) | 0.325 |
| CMR findings | | | | | | |
| LVEDVi (mL/m^2^) | 81 (72-90) | 114 (108-121) | **<0.001** | 91 (77-97) | 133 (119-152) | **<0.001** |
| LVEF (%) | 59 (52-64) | 52 (49-55) | **<0.001** | 40 (35-44) | 32 (25-39) | **<0.001** |
| LVMI (g/m^2^) | 62 (55-72) | 77 (63-88) | **<0.001** | 71 (56-84) | 86 (69-101) | **<0.001** |
| LV wall motion abnormalities, n (%) | 60 (29) | 33 (64) | **<0.001** | 29 (97) | 96 (97) | 0.933 |
| LGE presence in the LV, n (%) | 158 (76) | 38 (73) | 0.585 | 27 (90) | 71 (72) | **0.040** |
| n of LV segments with LGE, n | 2 (1-4) | 2 (0-6) | 0.257 | 3 (1-7) | 4 (0-7) | 0.665 |
| Percent LGE mass (% of LV mass) | 4 (0-8) | 4 (0-10) | 0.741 | 5 (3-15) | 6 (0-16) | 0.615 |
| Fatty replacement in the LV, n (%) | 114 (55) | 18 (35) | **0.007** | 11 (37) | 19 (19) | 0.051 |
| n of LV segments with fatty replacement, n | 1 (0-2) | 1 (0-2) | 0.837 | 3 (2-5) | 0 (0-1) | **0.019** |
| RVEDVi (mL/m^2^) | 80 (69-92) | 99 (88-114) | **<0.001** | 65 (57-69) | 80 (69-94) | **<0.001** |
| Moderate-to-severe RV dilation, n (%) | 0 (0) | 4 (8) | **<0.001** | 0 (0) | 2 (2) | 0.438 |
| RV wall motion abnormalities, n (%) | 108 (52) | 19 (37) | **0.044** | 5 (17) | 28 (28) | 0.192 |
| RVEF (%) | 58 (52-64) | 60 (56-63) | 0.175 | 54 (48-57) | 52 (43-61) | 0.857 |
| RV LGE, n (%) | 32 (16) | 4 (8) | 0.136 | 3 (10) | 7 (7) | 0.599 |
| Fatty replacement in the RV, n (%) | 105 (51) | 8 (15) | **<0.001** | 3 (10) | 7 (7) | 0.574 |
| Definite biventricular ACM, n (%) | 27 (13) | 6 (12) | 0.771 | 4 (13) | 17 (17) | 0.618 |

Percentages were calculated out of available values. Significant p values are reported in bold. CMR, cardiovascular magnetic resonance; LGE, late gadolinium enhancement; LP/P, likely pathogenic or pathogenic; LVEDVi, left ventricular end-diastolic volume index; LVEF, left ventricular ejection fraction; LVMI, left ventricular mass index; NDLVC, non-dilated left ventricular cardiomyopathy; NT-proBNP, N-terminal pro-B-type natriuretic peptide; NYHA, New York Heart Association; RVEDVi, right ventricular end-diastolic volume index; RVEF, right ventricular ejection fraction; VUS, variant of unknown significance.

**Supplemental Table 3. Patient characteristics according to the left ventricular ejection fraction (LVEF) cut-off 40% and LV dilation.**

|  | **LVEF >40%**  **n=294** | | | **LVEF ≤40%**  **n=94** | | |
| --- | --- | --- | --- | --- | --- | --- |
|  | **NDLVC**  **n=221 (75%)** | **DCM**  **n=73 (25%)** | **p** | **NDLVC**  **n=16 (17%)** | **DCM**  **n=78 (83%)** | **p** |
| Age (years) | 55 (44-64) | 52 (38-59) | **0.014** | 63 (49-74) | 56 (47-63) | **0.059** |
| Female sex, n (%) | 78 (35) | 20 (27) | 0.215 | 3 (19) | 24 (31) | 0.333 |
| Family history of SCD, n (%) | 77 (35) | 17 (23) | 0.068 | 4 (25) | 19 (24) | 0.978 |
| NT-proBNP (ng/L) | 78 (38-168) | 188 (88-375) | 0.046 | 662 (289-1,598) | 648 (300-1,368) | 0.577 |
| NYHA class I/II, n (%) | 117/104 (53/47) | 6/67 (8/92) | **<0.001** | 10/6 (63/37) | 14/64 (18/82) | **<0.001** |
| Hypertension, n (%) | 64 (29) | 12 (16) | **0.038** | 4 (25) | 13 (17) | **0.035** |
| Diabetes, n (%) | 21 (10) | 5 (7) | 0.278 | 2 (13) | 3 (4) | **0.021** |
| Gene testing | | | | | | |
| Negative/VUS/LP or P variant, n (%) | 121/48/52 (55/22/24) | 40/13/20 (55/18/27) | 0.691 | 10/3/3 (63/19/19) | 54/8/16 (69/10/21) | 0.629 |
| LP or P variant in a desmosomal gene, n (%) | 29 (13) | 6 (8) | 0.262 | 2 (13) | 2 (3) | 0.073 |
| CMR findings | | | | | | |
| LVEDVi (mL/m^2^) | 82 (72-90) | 117 (110-123) | **<0.001** | 94 (85-98) | 142 (120-164) | **<0.001** |
| LVEF (%) | 57 (51-64) | 50 (44-54) | **<0.001** | 36 (31-39) | 30 (23-35) | **0.007** |
| LVMI (g/m^2^) | 62 (55-72) | 77 (65-87) | **<0.001** | 77 (64-90) | 87 (70-104) | 0.073 |
| LV wall motion abnormalities, n (%) | 74 (34) | 53 (73) | **<0.001** | 15 (94) | 76 (97) | 0.445 |
| LGE presence in the LV, n (%) | 170 (77) | 53 (73) | 0.417 | 15 (94) | 56 (72) | 0.063 |
| n of LV segments with LGE, n | 2 (1-4) | 2 (0-6) | 0.217 | 4 (2-11) | 4 (0-6) | 0.219 |
| Percent LGE mass (% of LV mass) | 4 (0-9) | 4 (0-11) | 0.561 | 6 (3-19) | 6 (0-16) | 0.516 |
| Fatty replacement in the LV, n (%) | 119 (54) | 26 (36) | **0.006** | 6 (38) | 11 (14) | **0.029** |
| n of LV segments with fatty replacement, n | 1 (0-2) | 1 (0-2) | 0.485 | 3 (1-10) | 0 (0-1) | 0.164 |
| RVEDVi (mL/m^2^) | 79 (68-92) | 97 (85-111) | **<0.001** | 57 (54-69) | 77 (68-91) | **<0.001** |
| Moderate-to-severe RV dilation, n (%) | 0 (0) | 5 (7) | **<0.001** | 0 (0) | 1 (1) | 0.657 |
| RV wall motion abnormalities, n (%) | 111 (50) | 23 (32) | **0.005** | 2 (13) | 24 (31) | 0.130 |
| RVEF (%) | 58 (52-64) | 60 (55-63) | 0.280 | 54 (47-58) | 51 (42-60) | 0.787 |
| RV LGE, n (%) | 33 (15) | 6 (8) | 0.130 | 2 (13) | 5 (6) | 0.398 |
| Fatty replacement in the RV, n (%) | 106 (48) | 11 (15) | **<0.001** | 2 (13) | 4 (5) | 0.279 |
| Definite biventricular ACM, n (%) | 28 (13) | 8 (11) | 0.699 | 3 (19) | 15 (19) | 0.964 |

Percentages were calculated out of available values. Significant p values are reported in bold. CMR, cardiovascular magnetic resonance; LGE, late gadolinium enhancement; LP/P, likely pathogenic or pathogenic; LVEDVi, left ventricular end-diastolic volume index; LVEF, left ventricular ejection fraction; LVMI, left ventricular mass index; NDLVC, non-dilated left ventricular cardiomyopathy; NT-proBNP, N-terminal pro-B-type natriuretic peptide; NYHA, New York Heart Association; RVEDVi, right ventricular end-diastolic volume index; RVEF, right ventricular ejection fraction; VUS, variant of unknown significance.

**Supplemental Table 4. Outcome predictors in patients with non-dilated left ventricular cardiomyopathy (NDLVC).**

|  | **HR** | **95% CI** | **p** |
| --- | --- | --- | --- |
| Age (years) | - | - | 0.175 |
| Female sex | - | - | 0.873 |
| Family history of SCD | - | - | 0.916 |
| NT-proBNP (ng/L) | - | - | 0.475 |
| NYHA class I/II | - | - | 0.322 |
| Hypertension | - | - | 0.221 |
| Diabetes | - | - | 0.169 |
| Gene testing | | | |
| Negative/VUS/LP or P variant | - | - | 0.794 |
| LP or P variant in a desmosomal gene | - | - | 0.718 |
| CMR findings | | | |
| LVEDVi (mL/m^2^) | - | - | 0.719 |
| LVEF (%) | 0.94 | 0.90-0.99 | **0.010** |
| LVMI (g/m^2^) | - | - | 0.203 |
| LV wall motion abnormalities, n (%) | - | - | 0.113 |
| LGE presence in the LV | - | - | 0.470 |
| n of LV segments with LGE | - | - | 0.972 |
| Percent LGE mass (% of LV mass) | - | - | 0.464 |
| Fatty replacement in the LV | - | - | 0.755 |
| n of LV segments with fatty replacement | - | - | 0.254 |
| RVEDVi (mL/m^2^) | - | - | 0.209 |
| Moderate-to-severe RV dilation | - | - | - |
| RV wall motion abnormalities | - | - | 0.098 |
| RVEF (%) | - | - | 0.321 |
| RV LGE, n (%) | - | - | 0.343 |
| Fatty replacement in the RV | - | - | 0.954 |
| Definite biventricular ACM | - | - | 0.768 |

Results of univariable Cox regression analysis are reported. The significant p value is reported in bold. CI, confidence interval; CMR, cardiovascular magnetic resonance; HR, hazard ratio; LGE, late gadolinium enhancement; LP/P, likely pathogenic or pathogenic; LVEDVi, left ventricular end-diastolic volume index; LVEF, left ventricular ejection fraction; LVMI, left ventricular mass index; NT-proBNP, N-terminal pro-B-type natriuretic peptide; NYHA, New York Heart Association; RVEDVi, right ventricular end-diastolic volume index; RVEF, right ventricular ejection fraction; VUS, variant of unknown significance.

**Supplemental Figure 1. Flowchart of patient selection.**


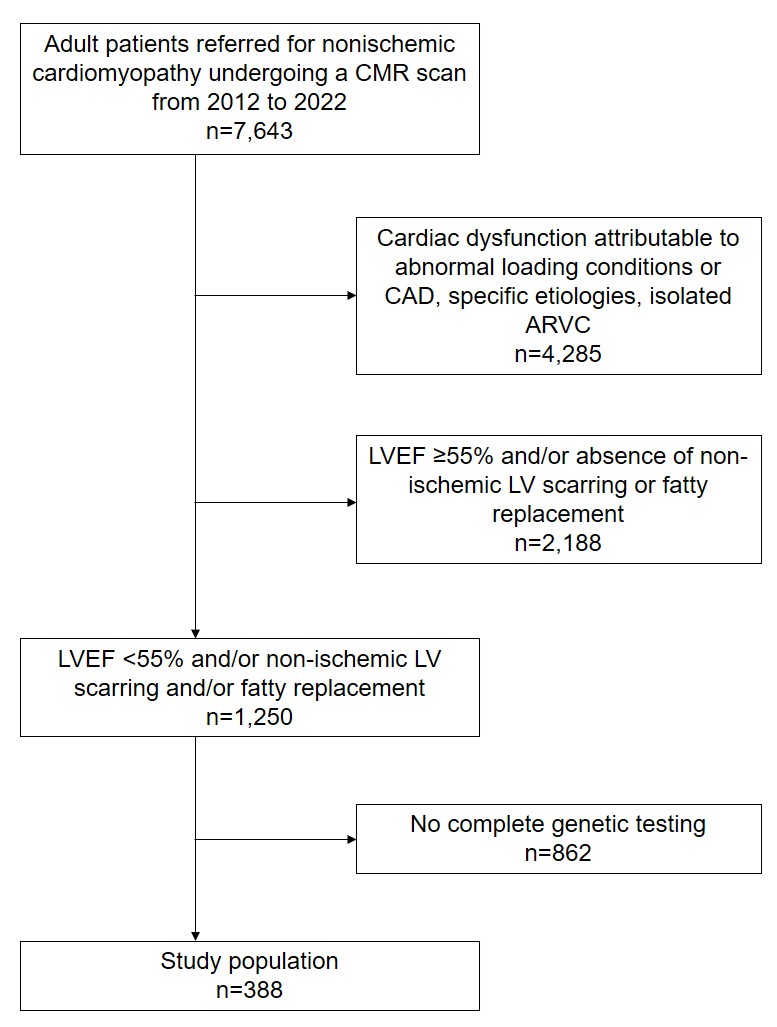


CMR, cardiovascular magnetic resonance; LV(EF), left ventricular (ejection fraction).

**Supplemental Figure 2. Left ventricular end-diastolic volume index (LVEDVi) and survival: spline curve analysis.**


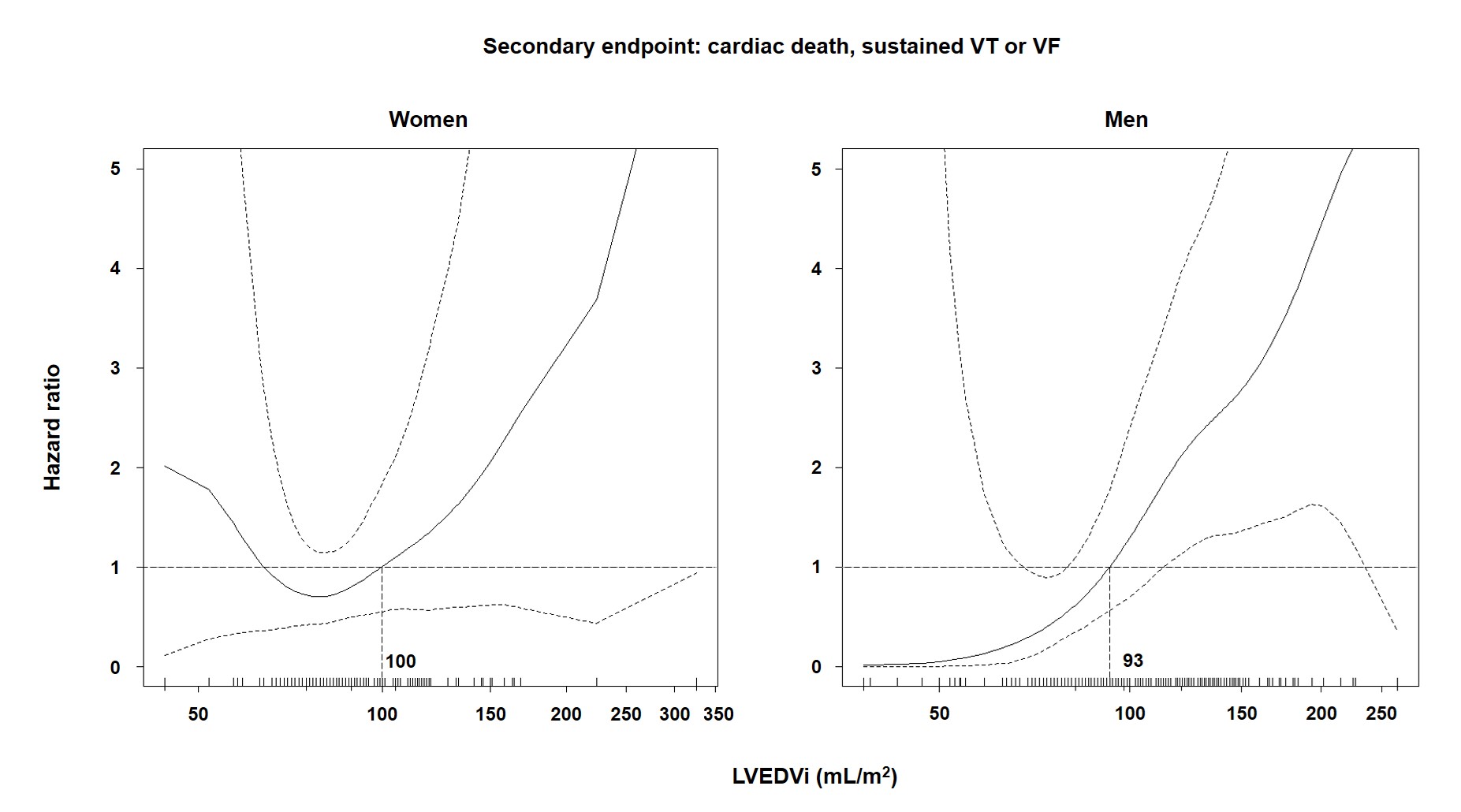


VF, ventricular fibrillation; VT, ventricular tachycardia.

**Supplemental Figure 3. Patients with no left ventricular (LV) dilation: LV ejection fraction (LVEF) cut-offs and secondary endpoint events.**


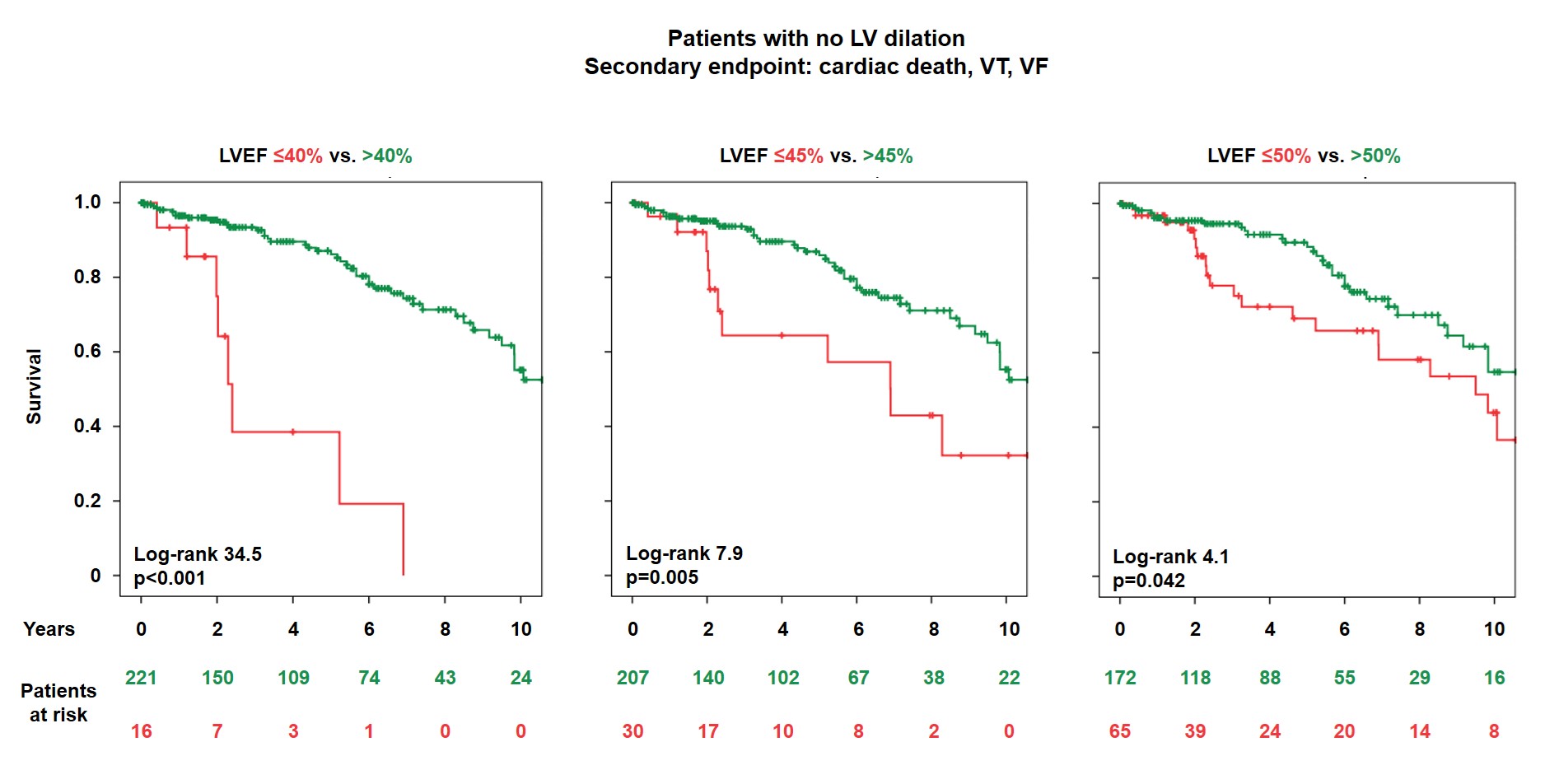
Kaplan-Meier survival curves. LVEDVi, LV end-diastolic volume index; M, men; W, women.

**Supplemental Figure 4. Survival according to left ventricular (LV) dilation and LV ejection fraction (LVEF) cut-offs: secondary endpoint.**


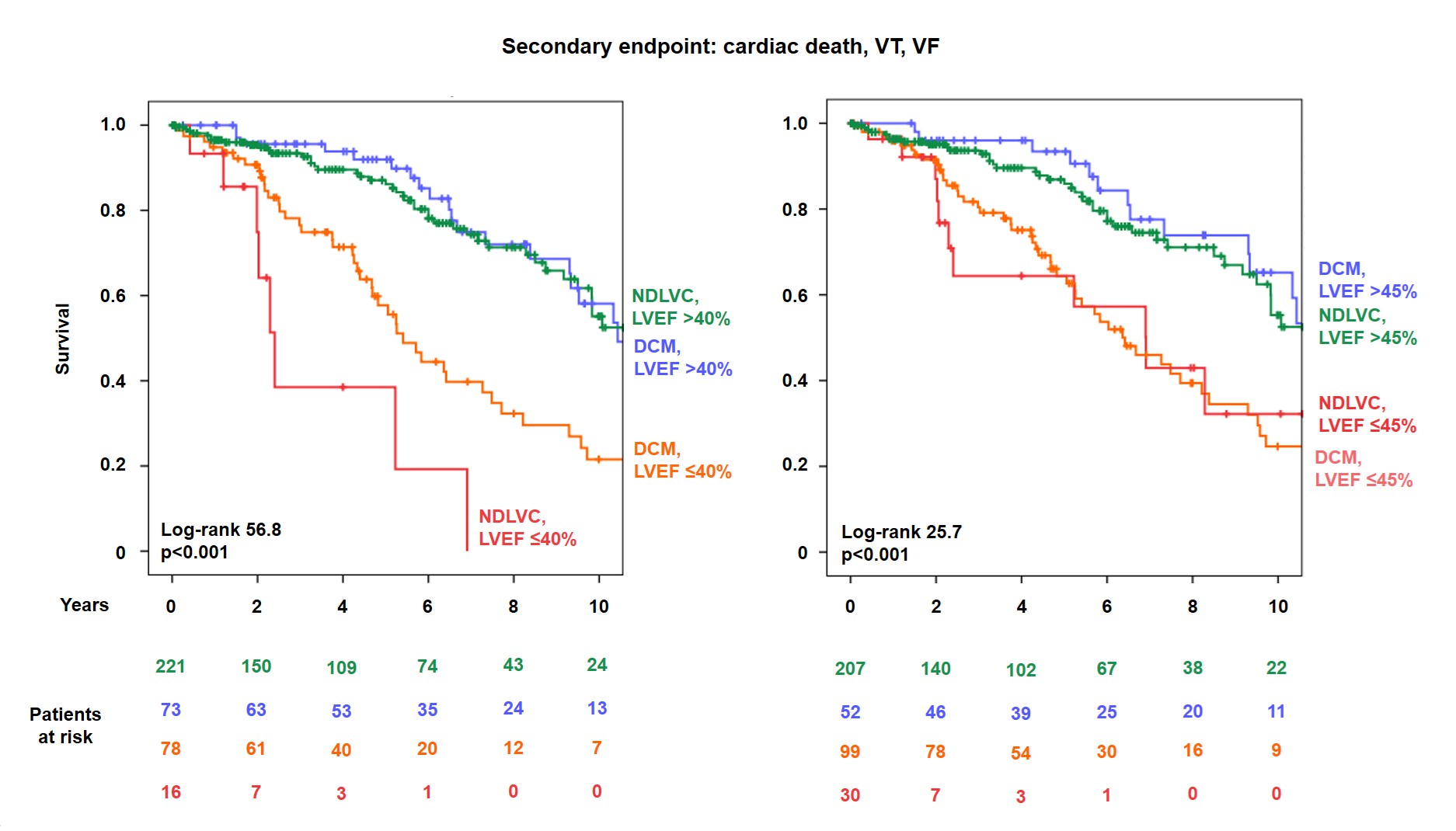


DCM, dilated cardiomyopathy; NDLVC, non-dilated left ventricular cardiomyopathy.
